# Time to Surgery Reduction in Hip Fracture Patients on an Integrated Orthogeriatric Unit: A Comparative Study of Three Healthcare Models

**DOI:** 10.1111/os.12633

**Published:** 2020-03-13

**Authors:** Carmen Pablos‐Hernández, Alfonso González‐Ramírez, Carmen da Casa, Maria Margarida Luis, María Aránzazu García‐Iglesias, Jose Miguel Julián‐Enriquez, Emiliano Rodríguez‐Sánchez, Juan F Blanco

**Affiliations:** ^1^ Unidad de Ortogeriatría Hospital Universitario de Salamanca Salamanca Spain; ^2^ Instituto de Investigación Biomédica de Salamanca (IBSAL) Salamanca Spain; ^3^ Servicio de Medicina Interna Centro Hospitalario de Vila Nova de Gaia Espinho Portugal; ^4^ Servicio de Admisión y Documentación Clínica Hospital Universitario de Salamanca Salamanca Spain; ^5^ Servicio de Traumatología y Cirugía Ortopédica Hospital Universitario de Salamanca Salamanca Spain; ^6^ Unidad de Investigación en Atención Primaria Salamanca Spain

**Keywords:** Healthcare model, Hip fractures, outcome and process assessment, health care, Time to surgery, Time to surgery, Time‐to‐treatment, Time management

## Abstract

**Objective:**

To investigate the clinical efficacy of three different healthcare models (Traditional Model, Geriatric Consultant Model, and Orthogeriatric Unit Model) consecutively applied to a single academic center (University Hospital of Salamanca, Spain) for older hip fracture patients.

**Methods:**

We performed a retrospective study, including 2741 hip fracture patients older than 64 years, admitted between 1 January 2003 and 31 December 2014 to the University Hospital of Salamanca. Patients were divided into three groups according to the healthcare model applied. There were 983 patients on the Traditional Model, 945 patients on the Geriatric Consultant Model, and 813 patients on the Orthogeriatric Unit Model. We recorded age and gender of patients, functional status at admission (Barthel Index, Katz Index, and Physical Red Cross Scale), type of fracture, and intervention, and we analyzed the length of stay, time to surgery, post‐surgical stay, and in‐hospital mortality according to the healthcare model applied.

**Results:**

Hip fractures are much more frequent in women, and an increase in the average age of patients was observed along with the study (*P* < 0.001). The most common type of fracture in the three models studied was an extracapsular fracture, for which the most common surgical procedure used was osteosynthesis. On the functional status of patients, there were no differences on the ambulatory ability previous to fracture, measured by the Physical Red Cross Scale, and the percentage of patients with a slight dependence determined by the Barthel Index (>60) was similar in both groups, but considering the Katz Index, the percentage of patients with a high degree of independence (A‐B) was significantly higher for the group of patients treated on the Orthogeriatric Unit Model period (56%, *P* = 0.009).

The Orthogeriatric Unit Model registered the greatest percentage of patients undergoing surgery (96.1%, *P* < 0.001) and the greatest number of early surgical procedures (<24 h) (24.8%, *P* < 0.001). The orthogeriatric unit model showed the shortest duration of stay (9 days median), decreasing by one day in respect of each of the other models studied (*P* < 0.001). Time to surgery was also significantly reduced with the Orthogeriatric Unit Model (median of 3 days, *P* < 0.001). With regard to in‐hospital follow‐up, there was a reduction in in‐hospital mortality during the study period. We observed differences among the three healthcare models, but without statistical significance.

**Conclusions:**

The healthcare model based on an Orthogeriatric Unit seems to be the most efficient, because it reaches a reduction in time to surgery, with an increased number of patients surgically treated on in the first 24 h, and the greatest frequency of surgically‐treated patients.

## Introduction

Hip fracture in the older population is a significant and enfeebling condition. Hip fracture is responsible for substantial mortality and morbidity of patients, and thereby responsible for high sanitary and social costs around the world. The global number of hip fractures is expected to increase from 1.26 mn in 1990 to 4.5 mn by the year 2050^1^. Hip fracture in Spain also remains one of the most important health problems in older patients^2^. In our province (Salamanca), several studies over the years have also demonstrated a high increasing incidence of hip fracture^3^.

To address this serious health problem, some initiatives have been launched. One relevant initiative has been the implementation of specific health care models, mainly the ones in which collaboration between surgeons and geriatricians or internists is established. These collaborative models of care can have a positive influence on the outcomes of hip fracture patients^4^, ^5^, ^6^, ^7^, since, in some cases, they are showing reduced mortality rates, reduced clinical complication rates, or being cost‐effective.

The high incidence, morbidity, and mortality associated with hip fractures have resulted in specific clinical models to facilitate patient care. A key factor for the adequate treatment of this outstanding pathology seems to be the interdisciplinary collaboration. In fact, the care model used may have relevance to the clinical outcomes^6^. Since the development of the Hastings care model^8^, we have learned that collaboration between the trauma team and the geriatric team may improve the treatment of patients with hip fractures. Several publications indicate that collaborative models between geriatricians and surgeons could improve the results of the treatment of this pathology^6^, ^7^, ^9^.

Some of these studies compare different models of care. These studies have demonstrated decreased length of in‐hospital stays, and thereby decreased costs, in addition to improvements in the functional results with collaborative models^10^. However, those improvements have not been demonstrated in other comparative works^11^. In any case, there seems to be a certain consensus, also in Spain, that collaborative care offers specific advantages to clinical care compared to other models^10^, ^12^.

The University Hospital of Salamanca (UHS) is a tertiary teaching center of the Spanish National Health System. Since the 1970s, three different healthcare models have been used in the UHS for older patients with hip fracture. The first model used was the so‐called Traditional Model (TM). In the TM, the traumatology team was leading the healthcare process, and the patient was evaluated by different specialists at the request of the traumatology team in determined clinical situations. In 2008, with the incorporation of a geriatrician specialist to the hospital staff, the first collaborative care model between geriatrics and traumatology was implemented: the Geriatric Consultant Model (GCM). In the GCM, the traumatology team was leading the healthcare process, and geriatrics intervene at the request of the traumatology team. The geriatric intervention allowed a better approach for the in‐hospitalized older hip fracture patients, assessing parameters that have not been assessed before. This collaborative model was implemented in one of the two orthopaedic and trauma services that coexisted in the UHS until 2013. With the unification of the two orthopaedic teams in 2013, the third and last healthcare model analyzed was implemented, the Orthogeriatric Unit Model (OUM). With this model, effective collaboration is established, with a shared responsibility between geriatrics and traumatology in the management of patients with hip fracture. Both are continuously taking care of and evaluating the clinical and functional status of every patient.

The purpose of this study was to determine if there was any continuous improvement in patient care as we progressed through each of the models. For this reason, we focus on the following points:

1. To analyze the functional status of hip fracture patients.
2. To analyze the mean length of stay (LOS) of patients in each period.
3. To analyze the in‐hospital mortality rates along with the study.

Those parameters and their further analysis would able us to emphasize our efforts on the best healthcare process. This study would be especially relevant in prioritizing patient care and being as cost‐effective as possible. There are no similar studies performed in our region, nor in Spain. The hip fracture reference health care model is not defined equally in all territories, and that could be a critical point due to the increasing incidence of hip fracture all over the word.

## Design and Methods

This is an observational epidemiological study, designed to compare three different healthcare models consecutively applied. The entire series of 2741 patients were managed in the same center with the three different protocols described, but with the same surgical, nursing, anesthesia, geriatric, and physiotherapy team.

The inclusion criteria are: (i) hip fracture patient admitted to the Trauma and Orthopaedics department of the University Hospital of Salamanca from 1 January 2003 to 31 December 2014; (ii) orthopaedic treatment or any surgical procedure (osteosynthesis‐based, partial or total hip replacement) for hip fracture treatment; (iii) the study population has to be stratified according to the care model used in each study period: (a) patients admitted from 1 January 2003 to 31 December 2007 were included in the TM group (983 patients); (b) patients admitted between 1 June 2008 and 1 May 2013 were included in the GCM group (945 patients); and, (c) patients admitted from 1 June 2013 to 31 December 2014 were included in the OUM group (813 patients); (iv) the major evaluation indications included the length of stay, time to surgery, post‐surgical hospital stay, and in‐hospital mortality rates stratified by the health care model applied; and (v) this is an observational epidemiological study, with retrospective design and descriptive character.

We have excluded all patients presenting pathological hip fracture or high energy trauma‐caused hip fracture.

## Data Collection

The population study included 2741 patients over 64 years of age who were stratified according to the care model used in each period (983 TM patients; 945 GCM patients; and 813 OUM patients). Data were obtained from the Minimum Basic Data Set (MBDS) coded by the UHS Clinical Documentation Unit, who registered both the main and secondary diagnoses as well as the surgical procedures and others following the ICD‐9 guidelines (100% codified).

For the present study, the following variables were collected:

*Biodemographic Variables*. We collected the gender of patients for a demographic approach along with the study. We also recorded the age (years) of every patient at admission, noting down the oldest patients aged 90 years or over.

*Fracture‐Related Variables*. We recorded the type of fracture, classifying it according to the standard division of hip fracture (intracapsular/extracapsular). Intracapsular fractures directly affect the head of the femur, while extracapsular fractures are defined around trochanters.

We also noted how many patients underwent an orthopaedic treatment or a surgical procedure. All surgical procedures were performed following neuraxial anesthesia of the patient.

The osteosynthesis procedure consisted of the use of a cephalous‐medullary nail. For this technique, we perform a mini‐approach on the top of the major trochanter. Hip replacement procedure has been used in two ways: hemiarthroplasty for patients with lower physical demands, and total hip replacement for active and younger patients. Hemiarthroplasty consisted of the use of a cemented femoral stent and a bipolar head. Total hip replacement consisted of the use of a cementless acetabular component. In both cases, we used a posterolateral approach to the hip.

*Length of Stay (LOS), Time to Surgery, and Post‐Surgical Stay*. The LOS was defined as the number of days a patient stayed in hospital, from admission to the Trauma and Orthopaedics department to discharge. We have also noted the time to surgery, defined as the number of days stayed in hospital previous to surgical procedure, noting down the patients who could undergo surgery in the first 24 h from admission. The post‐surgical stay was defined as the number of days stayed in hospital after the surgical procedure.

*Functional Status*. In collaborative care models (GCM and OUM) patients were evaluated through the Comprehensive Geriatric Assessment (CGA), assessing the patient's functional status at admission through the so‐called geriatric scores. In this case, the Barthel Index (BI), Katz Index (KI), and Physical Red Cross Scale (PRCS) have been used. These data are not available in the traditional healthcare model (TM) where there was no geriatric intervention.

*Barthel Index* *(BI)*. The BI, described by Mahoney and Barthel in 1965, collects data on the degree of capability for the development of 10 basic activities of daily living (ADL): feeding, bathing, grooming, dressing, toilet use, bowels, bladder, mobility, transfers, and stairs. For each activity analyzed, a gradual score is applied in five points, according to the patient ability to perform it, stratifying the patients into five categories: total dependence (<20 points), severe dependence (between 20 and 35 points), moderate dependence (between 40 and 55 points), slight dependence (between 60 and 95 points), or complete independence (100 points).

*Katz Index (KI)*. The KI, described in the 1960s by S. Katz for the evaluation of patients with hip fracture, estimates the independence of the patient to perform basic ADL, in a non‐gradual score. Specifically, it analyses six functions as independent or not independent, like feeding, bathing, dressing, toileting, continence, and transferring. The final result classifies patients from total independence named with the letter A to total dependence indicated with the letter G.

*Physical Red Cross Scale* *(PCRS)*. The PCRS score evaluates the physical ambulatory ability of the patient. It was developed at the Red Cross Hospital in Madrid in the 1970s and nowadays its use is declining due to the implementation of other non‐Spanish scores such as the Functional Ambulation Classification (FAC). The original Red Cross Scale has a mental‐status evaluation, not analyzed in our center, and a physical‐status evaluation, concerning five levels of ambulatory ability. Level 0 indicates full capability; level 1, some walking difficulty; level 2, instrumental aid for walking; level 3, personal aid; level 4, double personal aid; and level 5, any ambulatory capability (bed‐ridden).

*In‐hospital* *Mortality*. We recorded every patient being exitus previous to hospital discharge from the Trauma and Orthopaedics department.

## Data Analysis

Data were collected in a database formulated with Microsoft Excel processor and later imported into IBM® SPSS® Statics (ver. 23) program (New York, USA), which allowed statistical analysis to be carried out.

In the descriptive study, the quantitative variables are described with the mean and standard deviation. The qualitative variables are expressed by percentage and frequency.

For the comparative analysis, normal distribution (asymmetry and kurtosis) by Kolmogorov–Smirnov test of the quantitative variables was assessed, they were compared by nonparametric tests (Kruskal–Wallis test). Values reported have been represented by median and interquartile range. Comparison of qualitative variables was performed using the chi‐square association test (χ^2^). A *P*‐value less than 0.05 was considered statistically significant.

## Results

### *Biodemographic Variables*

Descriptive analysis of the study population, as well as data related to diagnosis and surgical procedure, are shown in Table 1. Hip fractures are much more frequent in women, a reality that is maintained in all three models, as only one in four patients were men in the study period overall. Regarding age, an increase in the average age of patients was observed along with the study (*P* < 0.001). At the initial study period, when the TM was implemented, the mean age was 84.23 years, while in the subsequent period, with the GCM, the mean age of the patient was 85.30 years, and in the most recent period, with the OUM, the mean age of patients was 85.73 years. The nonagenarian patients rise up to 31.6% in the most recent period (*P* = 0.010).

**Table 1:** Population characteristics

	Traditional model	Geriatric consultant model	Orthogeriatric unit model	*P* value
	(*n* = 983)	(*n* = 945)	(*n* = 813)	
**Gender**				0.428
Male	22.8% (*n* = 244)	24.6% (*n* = 232)	22.0% (*n* = 179)	
Female	77.2% (*n* = 759)	75.4% (*n* = 713)	78.0% (*n* = 634)	
**Age**				
Mean age (years)	84.23 ± 7.14	85.30 ± 6.86	85.73 ± 6.91	**<0.001**
Older than 90 years	25.1% (*n* = 247)	28.0% (*n* = 265)	31.6% (*n* = 257)	**0.010**
**Principal diagnosis**				0.468
Intracapsular fracture	48.7% (*n* = 479)	49.3% (*n* = 466)	46.5% (*n* = 378)	
Extracapsular fracture	51.3% (*n* = 504)	50.7% (*n* = 479)	53.5% (*n* = 435)	
**Type of intervention**				**<0.001**
Orthopedic treatment	10.0% (*n* = 98)	5.4% (*n* = 51)	3.9% (*n* = 32)	
Osteosynthesis	50.1% (*n* = 492)	52.8% (*n* = 499)	55.5% (*n* = 451)	
Partial hip replacement	38.7% (*n* = 380)	38.3% (*n* = 362)	39.6% (*n* = 322)	
Total hip replacement	1.3% (*n* = 13)	3.5% (*n* = 33)	1.0% (*n* = 8)	

Showing descriptive data of analyzed demographic variables, expressed by mean ± SD, or percentage and frequencies. *P*‐value of comparative analysis among groups is shown and significant differences are in bold.

### *Fracture‐Related Variables*

The most common type of fracture in the three models studied was an extracapsular fracture, for which the most common surgical procedure used was osteosynthesis (Table 1).

The percentage of patients whose hip fracture was treated surgically increased with the transition from one to the following model. Thus, of the patients admitted during the period in which the first model, TM, was utilized, 10% of patients were not treated surgically. This percentage was reduced by half with the implementation of the GCM (5.4%) and continued to decline with the implementation of the OUM (3.9%), showing these differences statistically significance (*P* < 0.001).

### *Functional Status at Admission*

Considering CGA performed, we could only analyze the two models implementing orthogeriatric collaboration. It was observed that, in the case of the BI, the percentage of patients with a slight dependence (BI > 60) was similar in both groups (BI 60: GCM = 80.2%, OUM = 81.3% [*P* = 0.557]). However, in the case of KI, the percentage of patients with a high degree of independence (IK A‐B) was higher for the group of patients treated with the OUM, and this difference was statistically significant (KI [AB]: GCM = 49.7%, OUM = 56.0% [*P* = 0.009]). Finally, analyzing the ability to ambulate prior to fracture according to the PRCI, the percentages of patients with a high ambulatory disability or totally incapacitated (physical red cross index; PCRI 4‐5) did not show statistically significant differences between the two models (PCRI [4‐5]: GCM = 4.5%; OUM = 6.2% [*P* = 0.129]).

### *Length of Stay, Time to Surgery, and Post‐Surgical Stay*

Regarding the analysis of the whole hospital stay, and pre‐ and postsurgical stays, as well as the percentage of patients undergoing surgery in the first 24 h, results are shown in Table 2.

**Table 2:** Stays and in‐hospital mortality

	Traditional model	Geriatric consultant model	Orthogeriatric unit model	*P* value
Length of stay (days)	10 [8,12]	11 [8,14]	9 [7,11]	**<0.001**
Time to surgery (days)	3 [2,5]	4 [2,5]	3 [1,5]	**<0.001**
Post‐surgical hospital stay (days)	7 [5,8]	7 [6,9]	6 [5,7]	**<0.001**
Early surgery (24 h)	5.1% (*n* = 50)	6.7% (*n* = 63)	24.8% (*n* = 202)	**<0.001**
In‐hospital mortality	5.1% (*n* = 50)	4.7% (*n* = 45)	3.4% (*n* = 28)	0.217

Showing descriptive data of hospital stay‐derived variables, expressed by median and interquartile range, or percentage and frequency. *P*‐values of comparative analysis are shown and significant differences are shown in bold.

The length of stay (LOS) of patients treated in the OUM was less than LOS of the other two groups, this difference was statistically significant (*P* < 0.001). The median LOS in the TM period was 10 days, which increased during the GCM period to 11 days and finally, was reduced to 9 days on the OUM period.

Time to surgery was also lower in this model (OUM), represented by a median of 3 days, and was statistically significant (*P* < 0.001). Regarding the number of patients who underwent surgery in the first 24 h after admission, we showed that approximately one in 20 TM patients could undergo surgery within the first 24 h, increasing this ratio to one in 15 patients at the GCM. This increase was much more noticeable with the implementation of OUM, with one in four patients undergoing surgery on in the first 24 h (Table 2). These differences were also statistically significant (*P* < 0.001).

Analyzing post‐surgical hospital stay, we also found statistically significant differences among the models (*P* < 0.001), which was also reduced in the OUM from 7 to 6 days.

### *In‐hospital Mortality*

With regard to in‐hospital mortality, there was a reduction in mortality during the study period. We observed differences among the three healthcare models, but without statistical significance (Table 2, *P* = 0.217).

## Discussion

In our center, due to its characteristics, three consecutive models of attention have been used and are analyzed in this work. These three models studied include a similar number of patients, whose average age has been increasing during the 12 years covered by the study and is similar to that of other published series^10^, ^12^. In the systematic review by Grigoryan *et al*. the average age of the patients in the series studied was slightly lower than ours, with the exception of one Spanish study. Similarly, the most common type of fracture in the three models analyzed was the extracapsular fracture. Several studies published over past decades have shown that extracapsular fractures affect older people, with low functional status and greater dependence on activities of daily living^13^. This fact must be taken into account when analyzing some works.

### *Functional Status*

Only in the models of collaboration between traumatology and geriatrics (GCM and OUM) has it been possible to have an assessment of the functional status of patients through the CGA. This improvement in functional status is an advantage of the collaborative models comparing to the non‐collaborative models, as already shown by Vidan *et al.* 14. The geriatric assessment is a quantitative indicator of the quality of care^15^ since it offers information that undoubtedly helps to provide better care. In our case, we have observed that in the models of collaboration, especially in the OUM where the geriatric intervention has been more present, the number of patients who received surgical treatment was significantly higher than the model where there was no geriatric intervention. Other studies show that those healthcare models, in which a geriatric team participates in the acute phase of patient care with hip fracture, obtain a higher rate of patients undergoing surgery^16^, ^17^. It could be deduced that the collaborative care models allow better clinical optimization of the patients to be submitted to surgical treatment. In our case, as shown in the results, the number of patients who received surgical treatment was higher in the OUM, despite the fact that the age was higher and there was a greater proportion of nonagenarians. We felt that this increase in surgically treated patients, despite a higher age, was a result of the improvements in interdisciplinary patient care.

### *Length of Stay*

A topic studied in this work refers to the in‐hospital length of stay. Although LOS depends on several factors such as the outpatient care structure of each country or region, some authors find a reduction in hospital stay when collaborative models are used, similar to those shown in our study^18^. Kristensen *et al*. did not find differences regarding LOS, while Zeltzer *et al*.^19^ found a greater mean LOS with the collaborative model. In our country, Vidan *et al*.^14^ demonstrated that an intensive and early geriatric intervention reduces mortality and the number of clinical complications, but did not find a significant effect on the LOS. From an economic perspective, it seems that collaborative models can be cost‐effective^20^.

We believe, in our case, that the decrease in the LOS with the OUM compared with the preceding models may be due to two factors fundamentally: the high rate of surgery and the reduction of surgical delay. With regard to this last issue, there is a consensus that the surgical treatment of older patients with hip fracture should be performed as soon as possible^21^, ^22^. The organizational change that occurred as a result of the unification of the orthopaedic surgery and trauma service and the implantation of the OUM has allowed a higher rate of surgery in the first 24 h, reaching a ratio of one in four patients. This is, in our point of view, one of the most relevant facts observed in this study.

### *In‐hospital Mortality*

In‐hospital mortality is one of the most studied indicators. Several authors found a reduction in mortality in collaborative care models^4^, ^23^. In our study, we observed a reduction in the number of deaths in patients under collaborative models, but those differences did not demonstrate statistical significance. This fact was also described in our country by Vidan *et al.* 14. The reduction of the in‐hospital mortality rate could be due to different causes. The decrease in the number of patients not treated surgically may be one of the influencing factors^14^, ^24^, and some publications consider mortality associated with hip fractures directly related to early surgical treatment^25^, ^26^, ^27^. However, Kristensen *et al.* 4, in a comparative study of OUM and TM, described a reduction in mortality independent of the surgical delay, and Lund *et al*.^28^ reported that neither the surgical delay nor the duration of the intervention were statistically significant risk factors for mortality after hip fracture surgery.

### *Limitations*

This study has some limitations. On the one hand, it is a retrospective study, and although the number of patients studied is large, variables studied are limited to data collected. Another limitation in our work is the lack of CGA in the TM, making it impossible to compare the functional situation data of patients according to the BI, KI, and PCRI. It would be interesting to perform prospective studies in hospitals where the OUM is not implemented yet, and more specific variables would be recorded for the outcome measurements.

### *Conclusions*

In view of these results, we conclude that the OUM care model is a more efficient model of care for hip fracture patients at our institution. With this care model, we were able to surgically treat a higher number of patients. In addition, it entails a reduction in perioperative hospital stay in our setting. The rate of interventions in the first 24 h, as well as the reduction of surgical delay, is an indicator of the quality of care in this type of pathology that the OUM seems to favor.

## Authorship Declaration

All authors listed meet the authorship criteria according to the latest guidelines of the International Committee of Medical Journal Editors. All authors approved the final version of the manuscript.
